# Association of patient- and hospital-level predictors with patterns of initial treatment in patients with rheumatoid arthritis: findings from a national cohort study

**DOI:** 10.1093/rheumatology/keae717

**Published:** 2024-12-24

**Authors:** Zijing Yang, Edward Alveyn, Mark Russell, Katie Bechman, Callum Coalwood, Elizabeth Price, Abhishek Abhishek, Sam Norton, James Galloway

**Affiliations:** Department of Inflammation Biology, Centre for Rheumatic Diseases, King’s College London, London, UK; Department of Inflammation Biology, Centre for Rheumatic Diseases, King’s College London, London, UK; Department of Inflammation Biology, Centre for Rheumatic Diseases, King’s College London, London, UK; Department of Inflammation Biology, Centre for Rheumatic Diseases, King’s College London, London, UK; British Society for Rheumatology, Bride House, London, UK; Department of Rheumatology, Great Western Hospital NHS Foundation Trust, Swindon, UK; The University of Nottingham, Nottingham NIHR-BRC, Nottingham, UK; Department of Inflammation Biology, Department of Psychology, Institute of Psychiatry, Psychology and Neuroscience, Centre for Rheumatic Diseases, King’s College London, London, UK; Department of Inflammation Biology, Centre for Rheumatic Diseases, King’s College London, London, UK

**Keywords:** rheumatoid arthritis, conventional synthetic disease-modifying antirheumatic drugs, methotrexate, treatment strategy

## Abstract

**Objectives:**

To update the first-line conventional synthetic DMARDs (csDMARDs) prescribing pattern, describe change and variation across demographical and geographical factors in the RA population, and identify individual and hospital factors associated with it.

**Methods:**

This retrospective cohort study included newly diagnosed RA adult patients from 1 May 2018 to 1 April 2023 in the UK. We used adjusted multinomial logistic regression with random effect to explore associations with different first-line csDMRAD prescription and to account for hospital-level clustering.

**Results:**

We identified 15 462 RA patients who received csDMARD treatment. Overall, 57% received MTX monotherapy and 14% received MTX combination therapy as first-line treatment. MTX is the most frequently medication, following by HCQ and SSZ. Compared with non-MTX prescription, prescription of MTX monotherapy [adjusted odds ratio (aOR) 1.25 95% CI (1.22–1.29)] and MTX combination therapy [aOR 1.45 (1.38–1.52)] was significantly higher in patients with higher DAS28, but lower in the non-White individuals with comorbidities: lung disease, cancer, fracture and heart attack. Among those who received MTX, monotherapy is more likely be prescribed in patients with higher DAS28 [aOR 1.08 (1.05–1.11)] and without lung disease [aOR 0.5 (0.44–0.56)], compared with combination therapy. Around 20% of the variability in first-line csDMARD prescribing was attributed to the hospital level.

**Conclusion:**

In this cohort study of new-onset RA population, both individual- and institution-level variation in first-line csDMARD treatment strategy was evident. Gender, ethnicity, disease activity, and comorbidities, especially lung disease, were associated with disparities at the individual level.

Rheumatology key messages

- The gap between clinical guideline–recommended conventional synthetic (cs) DMARD strategy and prescribing behaviours in real practice persists.
- Evidence persists that historic beliefs regarding methotrexate and lung disease persist despite contemporary evidence.
- Although individual patient factors explain most variation in csDMARD prescribing, notable variation is explained by hospital- and clinician-level factors, highlighting the need for robust local pathways to ensure best care.

## Introduction

RA is a chronic autoimmune inflammatory disease that can lead to joint destruction and deformity, and is associated with a considerable disease burden [1]. According to the Global Burden of Diseases study, over 31 million individuals worldwide will be living with RA by 2050 [2]. Strong evidence has indicated that early treatment and aggressive treatment using a treat-to-target approach can reduce damage to the joints and improve outcomes [3–5]. With development of new DMARDs, especially biological and targeted synthetic DMARDs (b/tsDMARDs) [6], treatment options for RA have increased substantially in recent years. Evidence from clinical trials suggests that a first-line biologic is no more effective than first-line conventional synthetic disease-modifying antirheumatic drugs (csDMARD) treatment in newly diagnosed patients [7]. csDMARDs, most commonly MTX [8], remain the first-line recommended strategy for newly diagnosed RA patients due to their effectiveness, acceptable safety profiles and low cost [9–12].

Prior clinical guidelines recommended MTX being part of the first treatment strategy, either as monotherapy or in combination with other csDMARDs [13]; moreover, csDMARD combination therapy was recommended by some national societies as preferred initial therapy [14, 15]. However, as the evidence has evolved and more high-quality studies assessed optimal medication, there has been a recognition that not all patients need a csDMARD combination from the outset. In some previous trial and cohort studies, triple therapy (MTX + SSZ + HCQ) did not demonstrate superiority but rather was associated with more adverse events, leading to low persistence rates [16–18]. This shift in practice is reflected in more recent guidelines, such as in the UK (NICE 2018), USA (ACR 2015) [19] and Europe (EULAR 2022) [10]. The 2018 NICE was updated to support csDMARD monotherapy as a first-line treatment. The 2022 EULAR RA management guideline was updated to encourage prescription of MTX monotherapy as first-line treatment [10].

In routine practice, MTX was historically withheld from RA patients with lung disease as it was suspected as a causative agent in pulmonary fibrosis. Recent studies have refuted this causal association and suggested a potential protective effect in delaying onset of interstitial lung disease (ILD) [20, 21]. It is not clear how much clinicians’ concern influences their prescribing behaviour and whether new evidence of the association between MTX and ILD, and guidelines recommending MTX monotherapy as first-line treatment, have been embraced in routine rheumatological care.

Previous studies demonstrated waiting times for starting DMARD treatment have decreased in recent decades [22], describing the prescribing patterns of DMARDs and assessed factors that influence the delay in initiating DMARDs, especially in b/tsDMARD [23–25]. These findings highlighted gaps between the evidence base and clinical practice in the real world and considerable heterogeneity exists in prescribing behaviour in RA, leading to inequity in health outcomes [22, 25, 26]. Data focusing on prescribing of MTX as monotherapy *vs* in combination with other csDMARDs are limited. More updated evidence is scarce concerning patterns of first-line csDMARDs, especially MTX in early RA in daily practice. Even less is known regarding variation across demographic and geographical characteristics. Therefore, in this study, we aimed to address this evidence gap by describing prescribing patterns and identifying factors associated with choice of initial csDMARD therapy among patients undergoing RA, incorporating a wide range of patient- and hospital-level factors.

## Methods

### Data source

We used data from the National Early Inflammatory Arthritis Audit (NEIAA), a national database of patients with early inflammatory arthritis (EIA) in the UK. A total of 131 trusts/health boards and 188 hospitals supplying rheumatology care and treating patients with suspected EIA contributed data. Individual- and hospital-level data are collected from clinicians through an online portal, prompting users to complete mandatory fields and sense-checking the provided information. In this study, data were utilized from hospitals participating in NEIAA from 1 May 2018 to 1 April 2023. All hospital names were anonymized.

### Population

We included patients aged 18 years or older who had a diagnosis of RA and were eligible for follow-up. Then, we limited the population to those who have data on csDMARD therapy and started csDMARD therapy in the main analysis.

### Independent variables

For independent variables, we included patient-level and hospital-level characteristics. Patient-level predictors included age, region, gender, ethnicity, symptom duration, presence of comorbidities, work status, whether referred via the EIA pathway, serotype (seropositivity and seronegativity), smoking status (categorized as current smoker, ex-smoker and never-smoker), CS use, Index of Multiple Deprivation, baseline DAS28, being prescribed on the same day as diagnosis, waiting time for rheumatology assessment, waiting time for the commencement of DMARD therapy and the DMARD delay. A lower Index of Multiple Deprivation level denotes less social deprivation in that area. Comorbidities include diabetes, hypertension, heart attack, lung disease, fracture, cancer, stomach ulcers and depression. Waiting time for the primary care referral was defined as the number of days to be referred to a rheumatology service after first presentation in primary care. Waiting time for rheumatology assessment was calculated as the number of days from referral to the first clinical assessment in suspected EIA patients. Waiting time for the commencement of DMARD therapy was defined as the number of days from referral to the initiation of first offered DMARD. Baseline DAS28 was reported as both continuous and categorical variables (low: a present score of ≤3.2; moderate: a present score between 3.2 and 5.1; high: a present score of >5.1). Tender joint count, swollen joint count and ESR were used to calculate the DAS28 score [27]. Index year of drug prescribing was included to account for temporal trends.

Hospital-level characteristics include staff structure (number of consultants, number of training grades, number of nurses) and clinical structure (having an EIA referral pathway agreed with primary care; having dedicated EIA clinics; having a locally agreed EIA treatment pathway; having access to musculoskeletal physiotherapy; having access to musculoskeletal US; and having access to musculoskeletal US on the same day as the assessment; having shared care; having emergency access; having a telephone adviceline).

### Outcome

The outcome of interest was the first csDMARD (MTX, HCQ, SSZ and LEF) therapy decision. We classified the type of csDMARD prescribed in this period in mutually exclusive categories (primary outcome: MTX monotherapy *vs* MTX combination *vs* other csDMARD strategies without MTX; secondary outcome: any MTX *vs* other csDMARD strategies without MTX). We also described the glucocorticoid (GC) exposure.

### Data analysis

Participant characteristics were presented as medians and first to third quartiles (interquartile range, IQR) for continuous variables. For categorical variables, characteristics were reported as frequencies and percentages. Differences between groups were assessed with the χ^2^ test and Kruskal–Wallis tests.

To describe the trend of prescription patterns, we calculated the proportion of patients who received each csDMARD first prescription as defined above among the new-onset RA patients by diagnosis year, gender, age and geographical region.

To explore other risk factors for csDMARD use, we estimated a series of multinomial logistic mixed-effects regression models to analyse the individual separately and hospital-level factors associated with the prescription [28, 29]. Specifically, we applied two-level multinomial logistic regression with random intercept to account for the clustering of patients within hospitals. In the patient-level analysis, we included age and gender as covariates in all models and sequentially estimated models with other relevant predictors (i.e. age and gender-adjusted) where predictors were selected based on clinical expertise and a literature review. In the hospital-level analysis, we chose a priori to control for age, ethnicity, smoking status and gender (case-mixed model). Results of unadjusted models are shown in the supplementary material.

Subsequently, we repeated these logistic regressions to examine the association between patient-level and hospital-level factors and the secondary outcome (any MTX *vs* other csDMARD strategies without MTX). Unadjusted odds ratios (ORs), adjusted odds ratios (aOR) and 95% CIs were calculated for all estimates.

To quantify the variation in outcomes explained between hospitals, we inspected the intraclass correlation coefficient (ICC) derived from the variance component estimates of the random effects [30, 31]. Specifically, the ICC was calculated as the ratio of the between-hospital variance to the total variance on a scale from 0 to 1, which indicates the proportion of the total variation in the outcome accounted for by the hospitals.

Missing data for categorical variables were included in the other or unknown group. Missingness for continuous variables ranged from 0% to 4.4%, which was small, and all variables were under 5% missing. As only a small percentage of patients had missing data, which should not have a meaningful impact on either the estimates or their precision in a sample of the size used for the study, a complete case analysis was employed for all regression models in main analysis [32, 33]. In the sensitivity analysis, mixed-effects logistic regression models were computed using the imputed data. Multiple imputations (m = 5) using chained equations was applied to account for missing estimator values. Rubin’s rules were used to pool estimates.

Two-sided *P* < 0.05 was interpreted as statistically significant. Statistical analyses were conducted using R version 4.3.2 (R Project for Statistical Computing). This study follows the Strengthening the Reporting of Observational Studies in Epidemiology (STROBE) reporting guideline [34].

### Ethical approval

No informed patient consent was required, as NEIAA has permission from the Secretary of State for Health to collect data for the purposes of national audit. Ethical approval for the secondary use in NEIAA has been obtained (Clinical Advisory Group Reference: 19/CAG/0059; Research Ethics Committee reference: 19/EE/0082).

## Results

### Characteristics of the study population

There were 16 683 RA patients over 18 years enrolled in NEIAA and eligible for follow-up [median (IQR) age, 61 (49–71) years; 63% female] from 186 hospitals (172 from England, 14 from Wales) from 2018 to 2023. Regarding ethnicity distribution, 14 159 participants (85%) were White, 1294 (7.8%) were Asian and 438 (2.6%) were Black. Of these, 78% (13 092) were issued monotherapy csDMARD prescriptions, 14% (2370) had combination csDMARD prescriptions and 8% (1221) did not have csDMARD prescriptions in the first 3 months after diagnosis. About 80% of those starting csDMARD were prescribed steroids at the same time. Over 60% of these received GC in combination with csDMARD monotherapy. The clinical, demographic and prescription characteristics of patients and the hospital characteristics are summarized in Table 1 and in Supplementary Tables S1 and S2A and B, available at *Rheumatology* online.

**Table 1.** Baseline table for patient characteristic

Patient characteristic	Overall, *N* = 16 683	MTX monotherapy *N* = 8807	MTX combination *N* = 2139	Other csDMARD strategies without MTX, *N* = 4516	No csDMARD, *N* = 1221
Age, years^a^					
Median (IQR)	61 (49, 71)	61 (51, 72)	58 (49, 69)	61 (47, 72)	61 (49, 73)
<40, *n*/*N* (%)	2044/16 443 (12)	904/8686 (10)	274/2114 (13)	707/4446 (16)	159/1197 (13)
40–50, *n*/*N* (%)	2145/16 443 (13)	1113/8686 (13)	301/2114 (14)	582/4446 (13)	149/1197 (12)
50–60, *n*/*N* (%)	3641/16 443 (22)	2017/8686 (23)	559/2114 (26)	819/4446 (18)	246/1197 (21)
60–70, *n*/*N* (%)	3914/16 443 (24)	2111/8686 (24)	514/2114 (24)	1020/4446 (23)	269/1197 (22)
70–80, *n*/*N* (%)	3601/16 443 (22)	1999/8686 (23)	382/2114 (18)	957/4446 (22)	263/1197 (22)
>80, *n*/*N* (%)	1098/16 443 (6.7)	542/8686 (6.2)	84/2114 (4.0)	361/4446 (8.1)	111/1197 (9.3)
Gender, female, *n*/*N* (%)	10 562/16 683 (63)	5457/8807 (62)	1362/2139 (64)	2993/4516 (66)	750/1221 (61)
Ethnicity, *n*/*N* (%)^a^					
White	14 159/16 683 (85)	7686/8807 (87)	1782/2139 (83)	3680/4516 (81)	1011/1221 (83)
Asian	1294/16 683 (7.8)	543/8807 (6.2)	194/2139 (9.1)	468/4516 (10)	89/1221 (7.3)
Black	438/16 683 (2.6)	172/8807 (2.0)	79/2139 (3.7)	143/4516 (3.2)	44/1221 (3.6)
Mixed	81/16 683 (0.5)	33/8807 (0.4)	6/2139 (0.3)	37/4516 (0.8)	5/1221 (0.4)
Other	540/16 683 (3.2)	279/8807 (3.2)	61/2139 (2.9)	145/4516 (3.2)	55/1221 (4.5)
Smoking status, *n*/*N* (%)^a^					
Never smoked	7111/16 683 (43)	3739/8807 (42)	929/2139 (43)	1907/4516 (42)	536/1221 (44)
Current smoker	3183/16 683 (19)	1674/8807 (19)	450/2139 (21)	820/4516 (18)	239/1221 (20)
Ex-smoker	4853/16 683 (29)	2581/8807 (29)	598/2139 (28)	1344/4516 (30)	330/1221 (27)
Duration of symptoms, *n*/*N* (%)^a^					
<1 month	1317/16 576 (7.9)	705/8742 (8.1)	164/2129 (7.7)	360/4493 (8.0)	88/1212 (7.3)
1–3 months	5659/16 576 (34)	3066/8742 (35)	736/2129 (35)	1492/4493 (33)	365/1212 (30)
3–6 months	3976/16 576 (24)	2116/8742 (24)	538/2129 (25)	1038/4493 (23)	284/1212 (23)
6–12 months	3082/16 576 (19)	1634/8742 (19)	378/2129 (18)	829/4493 (18)	241/1212 (20)
1–5 years	2122/16 576 (13)	1023/8742 (12)	275/2129 (13)	657/4493 (15)	167/1212 (14)
>5 years	420/16 576 (2.5)	198/8742 (2.3)	38/2129 (1.8)	117/4493 (2.6)	67/1212 (5.5)
Comorbidity, *n*/*N* (%)					
Lung disease^a^	1887/16 663 (11)	800/8800 (9.1)	199/2139 (9.3)	753/4514 (17)	135/1210 (11)
Heart attack	956/16 663 (5.7)	507/8800 (5.8)	100/2139 (4.7)	276/4514 (6.1)	73/1210 (6.0)
Hypertension	3507/16 663 (21)	1887/8800 (21)	423/2139 (20)	959/4514 (21)	238/1210 (20)
Fracture^a^	367/16 663 (2.2)	173/8800 (2.0)	39/2139 (1.8)	129/4514 (2.9)	26/1210 (2.1)
Diabetes	1584/16 663 (9.5)	847/8800 (9.6)	190/2139 (8.9)	426/4514 (9.4)	121/1210 (10)
Cancer	690/16 663 (4.1)	369/8800 (4.2)	74/2139 (3.5)	190/4514 (4.2)	57/1210 (4.7)
Stomach ulcer	602/16 663 (3.6)	295/8800 (3.4)	79/2139 (3.7)	178/4514 (3.9)	50/1210 (4.1)
Depression	1229/16 663 (7.4)	637/8800 (7.2)	157/2139 (7.3)	344/4514 (7.6)	91/1210 (7.5)
Seropositive, *n*/*N* (%)	11 486/15 850 (72)	6067/8435 (72)	1615/2054 (79)	3099/4287 (72)	705/1074 (66)
Baseline DAS28^a^					
Median (IQR)	4.99 (3.97, 5.91)	5.07 (4.15, 5.93)	5.36 (4.44, 6.31)	4.71 (3.59, 5.72)	4.30 (3.18, 5.52)
Low, *n*/*N* (%)	2087/15 979 (13)	856/8451 (10)	160/2083 (7.7)	789/4341 (18)	282/1104 (26)
Moderate, *n*/*N* (%)	6415/15 979 (40)	3428/8451 (41)	723/2083 (35)	1812/4341 (42)	452/1104 (41)
High, *n*/*N* (%)	7477/15 979 (47)	4167/8451 (49)	1200/2083 (58)	1740/4341 (40)	370/1104 (34)
Assessment waiting time, median (IQR)	17 (10, 31)	17 (10, 30)	17 (10, 31)	18 (11, 33)	19 (11, 35)
Treatment waiting time, median (IQR)	36 (20, 66)	36 (20, 64)	35 (20, 66)	35 (19, 71)	
Prescribe at same day as diagnosis, *n*/*N* (%)^a^	9375/15 462 (61)	4911/8807 (56)	1276/2139 (60)	3188/4516 (71)	
With CS, *n*/*N* (%)^a^	13 084/16 560 (79)	7270/8772 (83)	1829/2127 (86)	3279/4505 (73)	706/1156 (61)

a *P*-value of Kruskal–Wallis rank sum test or Chi-squared test <0.5. csDMARD: conventional synthetic DMARD; IQR: interquartile range.

### Treatment patterns

**Figure 1.**
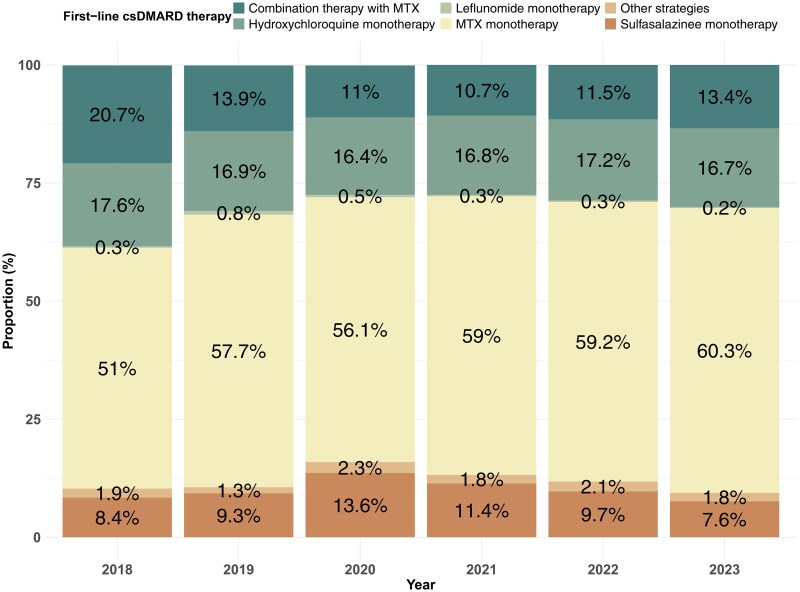
Trends of first-line conventional synthetic DMARD choice

**Figure 2.**
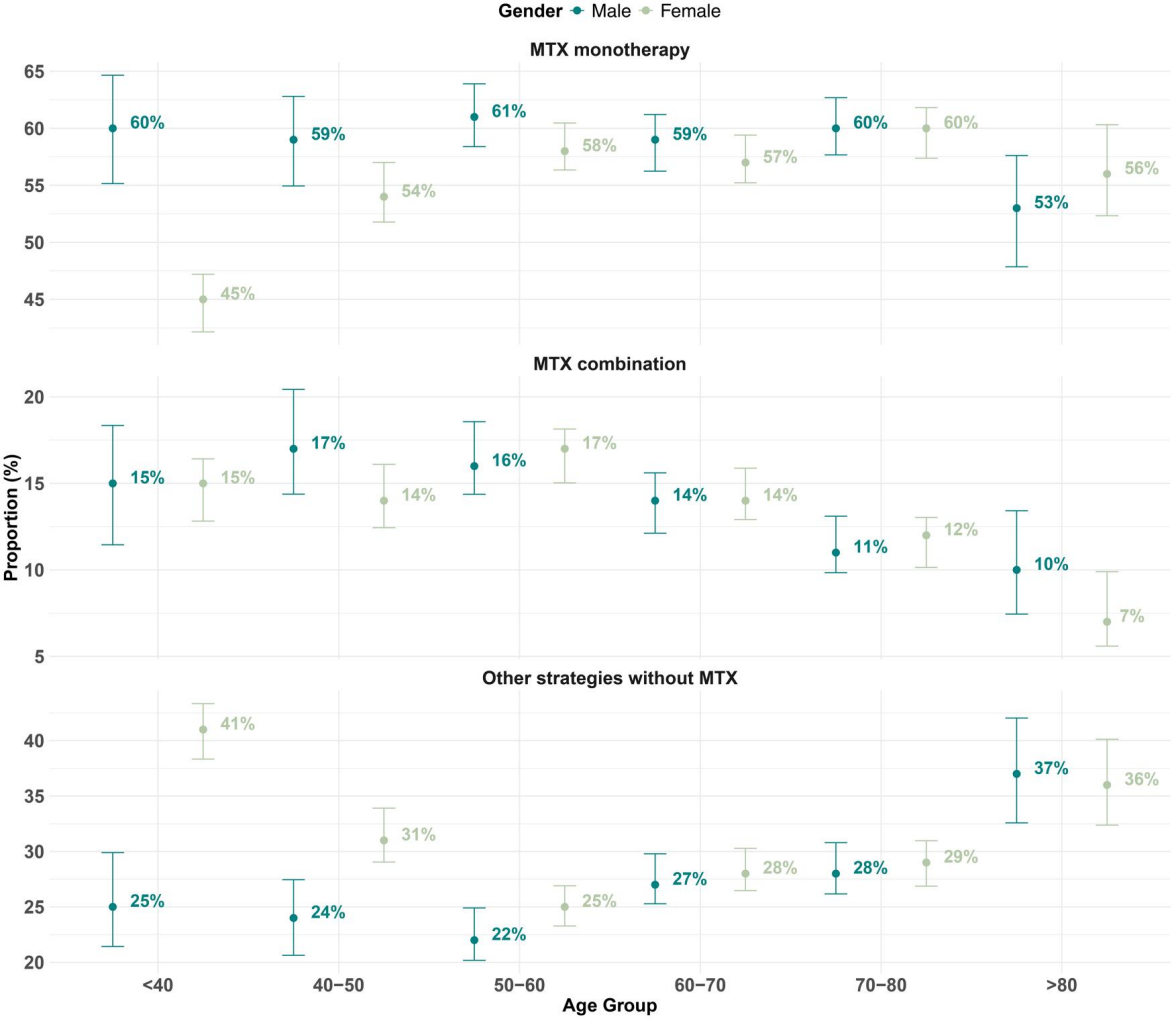
csDMARD strategy stratified by age and gender in RA patients with csDMARDs. csDMARD: conventional synthetic DMARD

Of these 15 462 patients receiving csDMARDs treatment, MTX was the most prescribed regimen [10 946/15 462; 70.8% (95% CI 70.1–71.5%)] followed by HCQ [4825/15 462; 31.2% (95% CI 30.5–31.9%)] and SSZ [1931/15 462; 12.5% (95% CI 12.0–13.5%)]. Around 80% of MTX prescriptions were given as monotherapy and 20% as part of combination therapy. Over the study period, the proportion of MTX monotherapy prescriptions increased modestly from 47% in 2018% to 56% in 2023. The internal pattern among all mono-csDMARD prescriptions is shown in Fig. 1, where MTX occupies a dominant position. Overall, 59.3% (95% CI 58.0–60.6%) of male and 55.6% (95% CI 54.6–56.6%) of female received the mono-MTX therapy. Patterns were broadly consistent when stratified by age and gender except in the subpopulation under 40 years old and the gap of gender difference in therapy narrowed over time (Fig. 2 and Supplementary Fig. S1, available at *Rheumatology* online). More information about csDMARD therapy stratification in subpopulations is shown in Supplementary Figs S1–S6, available at *Rheumatology* online.

### Patient-level factors associated with prescription

**Figure 3.**
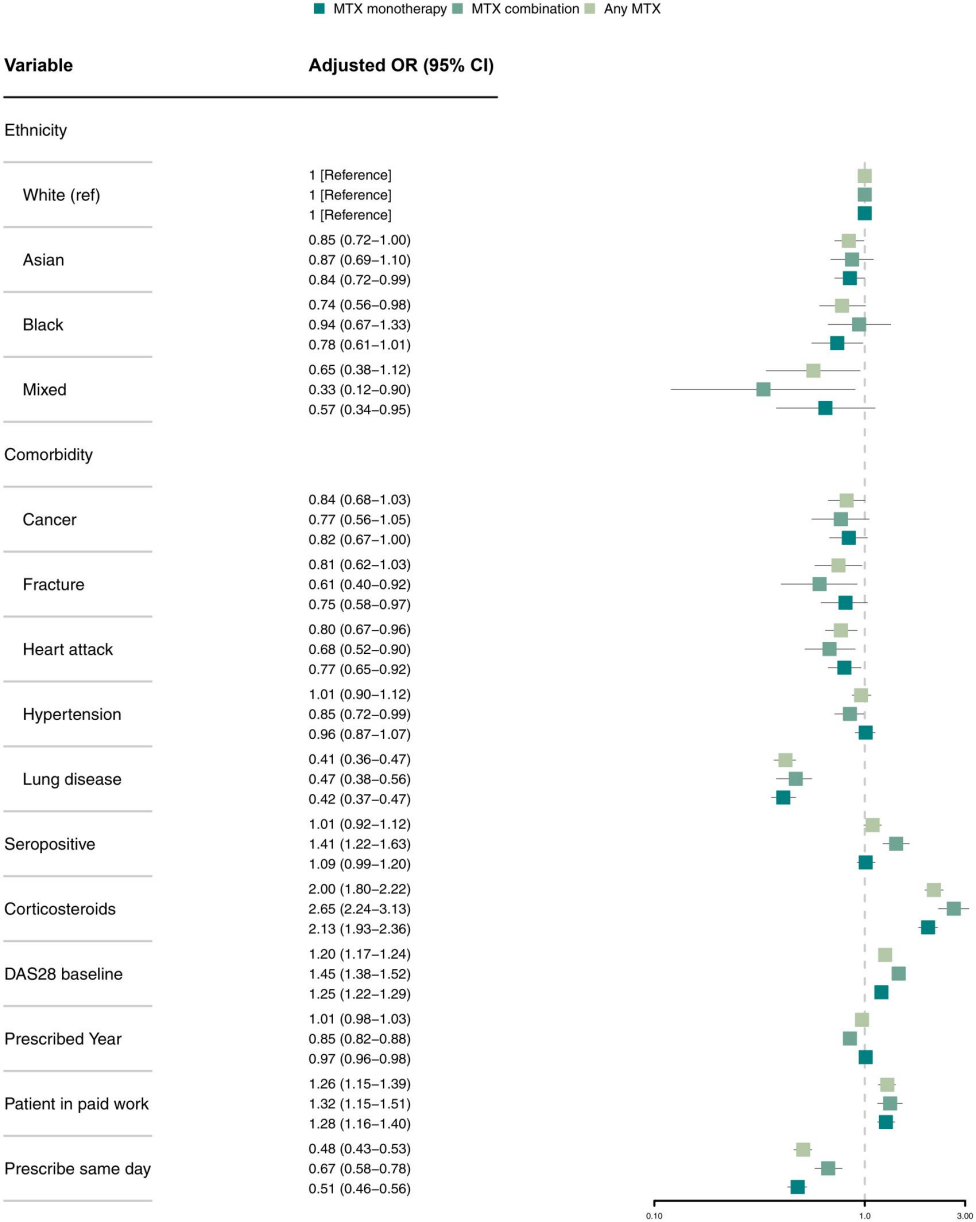
Forest plot summarizing the age-adjusted ORs for patient-level factors and MTX prescription compared with other conventional synthetic DMARD prescription. OR: odds ratio

Fig. 3 and Supplementary Table S3, available at *Rheumatology* online present the results of multilevel regression models which are adjusted by age and gender. Several patient characteristics were associated with MTX monotherapy prescribing in unadjusted model, and after adjusting for age and gender these associations persisted. Female gender and Asian and Black ethnicity were associated with a reduced likelihood of being prescribed MTX monotherapy. Patients with lung disease as a comorbidity were less frequently prescribed MTX [aOR of MTX monotherapy 0.41 (95% CI 0.36–0.47); MTX combination therapy: 0.47 (95% CI 0.38–0.56)]. Similar negative association is observed in patient with comorbidities including cancer, fracture history and previous myocardial infarction. Patients with higher disease activity had a higher likelihood [1.20 (95% CI 1.17–1.24)] of MTX monotherapy and combination therapy [1.45 (95% CI 1.38–1.52)], compared with other non-MTX strategies. In the subpopulation who were prescribed MTX, patients who were Black, with lung disease and lower DAS28 were less likely to receive MTX monotherapy compared with combination therapy (Supplementary Table S4, available at *Rheumatology* online). Results were similar after multiple imputation of missing data.

### Hospital-level factors associated with prescription

Disparities in csDMARD prescription proportion between regions decreased over time. The difference between the highest and the lowest regional proportion of MTX alone prescription reduced from 35% to 22% over the past 5 years. In general, there were significant improvements in MTX prescription in hospitals with musculoskeletal US [monotherapy: 1.47 (95% CI 1.11–1.95); combination therapy with MTX: 2.54 (95% CI 1.40–4.61)]. The association between MTX prescription and the availability of multi-disciplinary support including psychology, occupational therapy, podiatry and musculoskeletal physiotherapy is shown in Supplementary Table S5, available at *Rheumatology* online.

From the random-effects parameters in the multilevel logistic regression, the intraclass correlation coefficients indicated that 21% of the variability in initial MTX prescribing is accounted for by hospital-level characteristics that persisted after adjusting for patient characteristics.

## Discussion

This study provides insight into first-line treatment patterns among newly diagnosed RA patients and highlights important differences in treatment. Our results demonstrate that MTX treatment, especially monotherapy, is the most common strategy in line with guideline in routine practice and has become more predominant over time. Patient-level predictors explained the majority of variation in prescribing, and our data suggest that practice has not fully embraced the latest evidence. In parallel, our findings underscore the considerable variation in prescribing behaviour across hospitals, aligned to notable regional variation.

These temporal trends in the first csDMARD choice indicate an increasingly less aggressive approach to disease management in the UK. Nevertheless, an evidence–practice gap remains; the overall proportion of MTX prescriptions, especially in monotherapy, in our cohort remains lower than reported in Swedish and German cohorts [35, 36]. Our study identified only slight variations in MTX prescription patterns across age and gender groups, except in women under 40 years old. The potential reason for the observed lower proportion of younger female patients starting MTX treatment is likely explained by the recommendation to avoid MTX before a planned pregnancy [37]. Our findings are broadly consistent with the age and gender differences in treatment patterns observed in prior research, though differences appear smaller in magnitude [35, 36]. Reasons for age differences may include acceptance preferences [38, 39], prevalence of comorbidities for contraindications [38] and risk for infection across age groups [38]. Interestingly, despite difference in MTX prescription patterns across age groups, the MTX prescription rate was not substantially lower in the older population, especially those over 80 years old, even though they are at greater risk of drug–drug interactions and infection. This might be due to the relatively smaller sample size of this age group in our cohort. Strikingly, we observed considerable disparities in initiation of different first-line DMARD therapy across regions implying inequitable access among patients with new RA. Explanations likely include a combination of patient choice, patient comorbidities and clinician factors. Over 20% of the variability in MTX treatment was accounted for by institutional characteristics, suggesting a sizeable effect of clinician preference. This finding signals the importance of focusing on hospital-level differences to optimize the use of effective DMARD strategies nationally and ensure consistent provision of care across units.

Several factors associated with MTX prescription should be noted. Our study observed that although recent evidence has refuted the association between MTX exposure and ILD [20, 21], many clinicians are still reluctant to introduce MTX to patients with lung disease. This is a clinically important observation as it suggests that the stereotyped beliefs about MTX and ILD still persist in the rheumatology community. Additionally, we found a high rate of steroid prescriptions among individuals in our cohort (80%) and those who received MTX treatment (83%), which is higher than previous studies reported. A Swedish register-based study showed the treatment of around 70% of patients with GC [40], whereas this was only half of patients in a UK primary care-based cohort [41], although primary care records may well miss the very early period of treatment in RA. Both NICE and EULAR advocate the use of short-term GCs as a bridging therapy when starting csDMARD therapy with a rapid taper and discontinuation, but in the most recent update of the RA management guidelines from the ACR, the use of GCs was distinctly discouraged because of the toxicity risk.

According to our findings, if the csDMARD prescription occurred on the same day as diagnosis, the csDMARD chosen was less likely to be MTX. This delay may be due to awaiting additional test results that are needed before treatment, such as up-to-date laboratory results or a chest radiograph. In addition, in some departments it may be that patients are referred to nurses prior to prescribing in order to receive drug education, which may also delay treatment start. In previous studies exploring delays in starting DMARDs prescribing and the receipt of bDMARDs, clinician volume has shown an impact [36, 42, 43]. In our previous study, we noted the positive impact of hospital structure, such as access to musculoskeletal US, on the initiation of csDMARD prescriptions [43]. In the current study, we further observed the impact of hospital structure availability on MTX strategy.

### Strengths and limitations

This study is the first study to examine patterns of first-line csDMARD use on a national land regional level and is reflective of a new treatment landscape. Our results reflect prescribing practice in a realistic medical setting and add to the latest knowledge on how guideline recommendations on csDMARD treatment have been translated into actual practice in the UK. There are some limitations of our study. There are other unmeasured covariables (e.g. BMI, liver function tests) that we did not account for in this study that may affect DMARD prescription. We focused on the organizational-level variation, but individual clinician variation may also exist, even within the same hospital. Clinician variability and clinician-level characteristics and practices are not examined within this study, and may have contributed substantially to the observed institutional variation. This within-facility variation may be related to differences in training, personal attitudes toward the benefits and risks of drugs, and the way that individual physicians may respond to patient requests for specific medications. Our data quality also might be affected by selection and recall biases, as it relies on the contribution from clinicians’ reports.

## Conclusion

This study assessed the pattern of first-line csDMARD in RA patients. The finding suggests more MTX monotherapy is prescribed to RA patients now than in previous years. Differences in approach to treatment are associated with patient-, region- and hospital-level factors, and we present evidence that stereotyped decisions, such as regarding MTX and lung disease, persist. Our study also highlights the need for clinical and policy initiatives aimed at improving RA care pathways that focus on the hospital level.

## Supplementary Material

keae717_Supplementary_Data

## Data Availability

Data from the National Early Inflammatory Arthritis Audit used to produce this analysis is available on request, subject to the approval of the Healthcare Quality Improvement Partnership and the British Society for Rheumatology.
