# Supplementary material for: Association of patient- and hospital-level predictors with patterns of initial treatment in patients with rheumatoid arthritis: findings from a national cohort study
Source: Rheumatology (Oxford). 2024 Dec 24;64(6):3379–87. doi: 10.1093/rheumatology/keae717 (PMC12107036; doi:10.1093/rheumatology/keae717)
Supplement: keae717_Supplementary_Data [file keae717_supplementary_data.docx]

Table S1 Baseline table for patient characteristic

| Characteristic | Overall,  N = 16,683 | MTX monotherapy  N = 8,807 | MTX combination  N=2,139 | Other csDMARD strategies without MTX  N = 4,516 | No csDMARD,  N = 1,221 |
| --- | --- | --- | --- | --- | --- |
| **Patient Characteristic** |  |  |  |  |  |
| Age* |  |  |  |  |  |
| Median (IQR) | 61 (49, 71) | 61 (51, 72) | 58 (49, 69) | 61 (47, 72) | 61 (49, 73) |
| <40 | 2,044 / 16,443 (12%) | 904 / 8,686 (10%) | 274 / 2,114 (13%) | 707 / 4,446 (16%) | 159 / 1,197 (13%) |
| 40-50 | 2,145 / 16,443 (13%) | 1,113 / 8,686 (13%) | 301 / 2,114 (14%) | 582 / 4,446 (13%) | 149 / 1,197 (12%) |
| 50-60 | 3,641 / 16,443 (22%) | 2,017 / 8,686 (23%) | 559 / 2,114 (26%) | 819 / 4,446 (18%) | 246 / 1,197 (21%) |
| 60-70 | 3,914 / 16,443 (24%) | 2,111 / 8,686 (24%) | 514 / 2,114 (24%) | 1,020 / 4,446 (23%) | 269 / 1,197 (22%) |
| 70-80 | 3,601 / 16,443 (22%) | 1,999 / 8,686 (23%) | 382 / 2,114 (18%) | 957 / 4,446 (22%) | 263 / 1,197 (22%) |
| >80 | 1,098 / 16,443 (6.7%) | 542 / 8,686 (6.2%) | 84 / 2,114 (4.0%) | 361 / 4,446 (8.1%) | 111 / 1,197 (9.3%) |
| Gender-Female, n / N (%)* | 10,562 / 16,683 (63%) | 5,457 / 8,807 (62%) | 1,362 / 2139 (64%) | 2,993 / 4516 (66%) | 750 / 1,221 (61%) |
| Ethnicity, n / N (%)* |  |  |  |  |  |
| White | 14,159 / 16,683 (85%) | 7,686 / 8,807 (87%) | 1,782 / 2,139 (83%) | 3,680 / 4,516 (81%) | 1,011 / 1,221 (83%) |
| Asian | 1,294 / 16,683 (7.8%) | 543 / 8,807 (6.2%) | 194 / 2,139 (9.1%) | 468 / 4,516 (10%) | 89 / 1,221 (7.3%) |
| Black | 438 / 16,683 (2.6%) | 172 / 8,807 (2.0%) | 79 / 2,139 (3.7%) | 143 / 4,516 (3.2%) | 44 / 1,221 (3.6%) |
| Mixed | 81 / 16,683 (0.5%) | 33 / 8,807 (0.4%) | 6 / 2,139 (0.3%) | 37 / 4,516 (0.8%) | 5 / 1,221 (0.4%) |
| Other | 540 / 16,683 (3.2%) | 279 / 8,807 (3.2%) | 61 / 2,139 (2.9%) | 145 / 4,516 (3.2%) | 55 / 1,221 (4.5%) |
| Smoking status, n / N (%)* |  |  |  |  |  |
| Never smoked | 7,111 / 16,683 (43%) | 3,739 / 8,807 (42%) | 929 / 2,139 (43%) | 1,907 / 4,516 (42%) | 536 / 1,221 (44%) |
| Current smoker | 3,183 / 16,683 (19%) | 1,674 / 8,807 (19%) | 450 / 2,139 (21%) | 820 / 4,516 (18%) | 239 / 1,221 (20%) |
| Ex-smoker | 4,853 / 16,683 (29%) | 2,581 / 8,807 (29%) | 598 / 2,139 (28%) | 1,344 / 4,516 (30%) | 330 / 1,221 (27%) |
| In paid work, n / N (%)* | 7,338 / 16,451 (45%) | 3,907 / 8,679 (45%) | 1,057 / 2,106 (50%) | 1,885 / 4,469 (42%) | 489 / 1,197 (41%) |
| Duration of symptoms, n / N (%)* |  |  |  |  |  |
| <1 month | 1,317 / 16,576 (7.9%) | 705 / 8,742 (8.1%) | 164 / 2,129 (7.7%) | 360 / 4,493 (8.0%) | 88 / 1,212 (7.3%) |
| 1-3 months | 5,659 / 16,576 (34%) | 3,066 / 8,742 (35%) | 736 / 2,129 (35%) | 1,492 / 4,493 (33%) | 365 / 1,212 (30%) |
| 3-6 months | 3,976 / 16,576 (24%) | 2,116 / 8,742 (24%) | 538 / 2,129 (25%) | 1,038 / 4,493 (23%) | 284 / 1,212 (23%) |
| 6-12 months | 3,082 / 16,576 (19%) | 1,634 / 8,742 (19%) | 378 / 2,129 (18%) | 829 / 4,493 (18%) | 241 / 1,212 (20%) |
| 1-5 years | 2,122 / 16,576 (13%) | 1,023 / 8,742 (12%) | 275 / 2,129 (13%) | 657 / 4,493 (15%) | 167 / 1,212 (14%) |
| >5 years | 420 / 16,576 (2.5%) | 198 / 8,742 (2.3%) | 38 / 2,129 (1.8%) | 117 / 4,493 (2.6%) | 67 / 1,212 (5.5%) |
| Comorbidity |  |  |  |  |  |
| Lung disease, n / N (%)* | 1,887 / 16,663 (11%) | 800 / 8,800 (9.1%) | 199 / 2,139 (9.3%) | 753 / 4,514 (17%) | 135 / 1,210 (11%) |
| Heart attack, n / N (%) | 956 / 16,663 (5.7%) | 507 / 8,800 (5.8%) | 100 / 2,139 (4.7%) | 276 / 4,514 (6.1%) | 73 / 1,210 (6.0%) |
| Hypertension, n / N (%) | 3,507 / 16,663 (21%) | 1,887 / 8,800 (21%) | 423 / 2,139 (20%) | 959 / 4,514 (21%) | 238 / 1,210 (20%) |
| Fracture, n / N (%)* | 367 / 16,663 (2.2%) | 173 / 8,800 (2.0%) | 39 / 2,139 (1.8%) | 129 / 4,514 (2.9%) | 26 / 1,210 (2.1%) |
| Diabetes, n / N (%) | 1,584 / 16,663 (9.5%) | 847 / 8,800 (9.6%) | 190 / 2,139 (8.9%) | 426 / 4,514 (9.4%) | 121 / 1,210 (10%) |
| Cancer, n / N (%) | 690 / 16,663 (4.1%) | 369 / 8,800 (4.2%) | 74 / 2,139 (3.5%) | 190 / 4,514 (4.2%) | 57 / 1,210 (4.7%) |
| Stomach ulcer, n / N (%) | 602 / 16,663 (3.6%) | 295 / 8,800 (3.4%) | 79 / 2,139 (3.7%) | 178 / 4,514 (3.9%) | 50 / 1,210 (4.1%) |
| Depression, n / N (%) | 1,229 / 16,663 (7.4%) | 637 / 8,800 (7.2%) | 157 / 2,139 (7.3%) | 344 / 4,514 (7.6%) | 91 / 1,210 (7.5%) |
| Seropositive, n / N (%)* | 11,486 / 15,850 (72%) | 6,067 / 8,435 (72%) | 1,615 / 2,054 (79%) | 3,099 / 4,287 (72%) | 705 / 1,074 (66%) |
| Baseline DAS28* |  |  |  |  |  |
| Median (IQR) | 4.99 (3.97, 5.91) | 5.07 (4.15, 5.93) | 5.36 (4.44, 6.31) | 4.71 (3.59, 5.72) | 4.30 (3.18, 5.52) |
| Low, n / N (%) | 2,087 / 15,979 (13%) | 856 / 8,451 (10%) | 160 / 2,083 (7.7%) | 789 / 4,341 (18%) | 282 / 1,104 (26%) |
| Moderate, n / N (%) | 6,415 / 15,979 (40%) | 3,428 / 8,451 (41%) | 723 / 2,083 (35%) | 1,812 / 4,341 (42%) | 452 / 1,104 (41%) |
| High, n / N (%) | 7,477 / 15,979 (47%) | 4,167 / 8,451 (49%) | 1,200 / 2,083 (58%) | 1,740 / 4,341 (40%) | 370 / 1,104 (34%) |
| IMD, n / N (%)* |  |  |  |  |  |
| 1 (Least deprived) | 1,343 / 16,683 (8.1%) | 774 / 8,807 (8.8%) | 138 / 2,139 (6.5%) | 328 / 4,516 (7.3%) | 103 / 1,221 (8.4%) |
| 2 | 1,425 / 16,683 (8.5%) | 729 / 8,807 (8.3%) | 194 / 2,139 (9.1%) | 393 / 4,516 (8.7%) | 109 / 1,221 (8.9%) |
| 3 | 1,640 / 16,683 (9.8%) | 764 / 8,807 (8.7%) | 281 / 2,139 (13%) | 483 / 4,516 (11%) | 112 / 1,221 (9.2%) |
| 4 | 1,612 / 16,683 (9.7%) | 846 / 8,807 (9.6%) | 165 / 2,139 (7.7%) | 500 / 4,516 (11%) | 101 / 1,221 (8.3%) |
| 5 | 1,739 / 16,683 (10%) | 979 / 8,807 (11%) | 258 / 2,139 (12%) | 375 / 4,516 (8.3%) | 127 / 1,221 (10%) |
| 6 | 1,606 / 16,683 (9.6%) | 794 / 8,807 (9.0%) | 213 / 2,139 (10.0%) | 491 / 4,516 (11%) | 108 / 1,221 (8.8%) |
| 7 | 1,625 / 16,683 (9.7%) | 819 / 8,807 (9.3%) | 212 / 2,139 (9.9%) | 433 / 4,516 (9.6%) | 161 / 1,221 (13%) |
| 8 | 1,522 / 16,683 (9.1%) | 873 / 8,807 (9.9%) | 202 / 2,139 (9.4%) | 315 / 4,516 (7.0%) | 132 / 1,221 (11%) |
| 9 | 1,800 / 16,683 (11%) | 979 / 8,807 (11%) | 215 / 2,139 (10%) | 497 / 4,516 (11%) | 109 / 1,221 (8.9%) |
| 10 (Most) | 1,502 / 16,683 (9.0%) | 755 / 8,807 (8.6%) | 161 / 2,139 (7.5%) | 468 / 4,516 (10%) | 118 / 1,221 (9.7%) |
| Region, n / N (%) |  |  |  |  |  |
| EAST OF ENGLAND | 1,978 / 16,683 (12%) | 884 / 8,807 (10%) | 305 / 2,139 (14%) | 672 / 4,516 (15%) | 117 / 1,221 (9.6%) |
| LONDON | 1,869 / 16,683 (11%) | 665 / 8,807 (7.6%) | 348 / 2,139 (16%) | 679 / 4,516 (15%) | 177 / 1,221 (14%) |
| MIDLANDS | 3,225 / 16,683 (19%) | 1,598 / 8,807 (18%) | 466 / 2,139 (22%) | 872 / 4,516 (19%) | 289 / 1,221 (24%) |
| North East and Yorkshire | 2,452 / 16,683 (15%) | 1,339 / 8,807 (15%) | 240 / 2,139 (11%) | 670 / 4,516 (15%) | 203 / 1,221 (17%) |
| North West | 1,956 / 16,683 (12%) | 981 / 8,807 (11%) | 320 / 2,139 (15%) | 489 / 4,516 (11%) | 166 / 1,221 (14%) |
| South East | 2,317 / 16,683 (14%) | 1,441 / 8,807 (16%) | 192 / 2,139 (9.0%) | 507 / 4,516 (11%) | 177 / 1,221 (14%) |
| South West | 1,983 / 16,683 (12%) | 1,415 / 8,807 (16%) | 96 / 2,139 (4.5%) | 403 / 4,516 (8.9%) | 69 / 1,221 (5.7%) |
| Wales | 903 / 16,683 (5.4%) | 484 / 8,807 (5.5%) | 172 / 2,139 (8.0%) | 224 / 4,516 (5.0%) | 23 / 1,221 (1.9%) |
| Assessment Waiting time Median (IQR) | 17 (10, 31) | 17 (10, 30) | 17 (10, 31) | 18 (11, 33) | 19 (11, 35) |
| Treatment waiting time Median (IQR) | 36 (20, 66) | 36 (20, 64) | 35 (20, 66) | 35 (19, 71) | - |
| Referral via EIA pathway, n / N (%) | 11,837 / 16,540 (72%) | 6,340 / 8,734 (73%) | 1,441 / 2,121 (68%) | 3,228 / 4,478 (72%) | 828 / 1,207 (69%) |
| Prescribe at same day as diagnosis, n / N (%)* | 9,375 / 15,462 (61%) | 4,911 / 8,807 (56%) | 1,276 / 2,139 (60%) | 3,188 / 4,516 (71%) | - |
| With corticosteroids, n / N (%)* | 13,084 / 16,560 (79%) | 7,270 / 8,772 (83%) | 1,829 / 2,127 (86%) | 3,279 / 4,505 (73%) | 706 / 1,156 (61%) |

*P value of Kruskal-Wallis rank sum test; Pearson's Chi-squared test less than 0.5

Table S2a Hospital characteristic table

|  |  |  |  |  |  |
| --- | --- | --- | --- | --- | --- |
| **Hospital Characteristic** | Overall,  N = 16,683 | MTX monotherapy  N = 8,807 | MTX combination  N=2,139 | Other csDMARD strategies without MTX  N = 4,516 | No DMARD,  N = 1,221 |
| Number of WTE Consultant, Median (IQR) | 4.20 (3.00, 6.20) | 4.25 (3.00, 6.20) | 4.00 (2.90, 5.80) | 4.10 (2.80, 6.30) | - |
| Number of WTE Training Grade, Median (IQR) | 1.00 (0.50, 2.20) | 1.00 (0.80, 2.28) | 1.00 (0.60, 2.40) | 1.00 (0.50, 2.00) | - |
| Number of Rheumatology Nurses, Median (IQR) | 3.80 (2.80, 5.20) | 3.91 (3.00, 5.00) | 3.50 (2.50, 5.66) | 3.60 (2.50, 5.20) | - |
| Access Musculoskeletal Physio, n / N (%) | 12,009 / 13,240 (91%) | 6,817 / 7,520 (91%) | 1,637 / 1,811 (90%) | 3,555 / 3,909 (91%) | - |
| Access Podiatry, n / N (%) | 9,980 / 13,240 (75%) | 5,605 / 7,520 (75%) | 1,340 / 1,811 (74%) | 3,035 / 3,909 (78%) | - |
| Access Occupational Therapy, n / N (%) | 12,664 / 13,240 (96%) | 7,175 / 7,520 (95%) | 1,715 / 1,811 (95%) | 3,774 / 3,909 (97%) | - |
| Access Psychology, n / N (%) | 4,231 / 13,240 (32%) | 2,305 / 7,520 (31%) | 547 / 1,811 (30%) | 1,379 / 3,909 (35%) | - |
| Early Arthritis Pathway, n / N (%) | 10,859 / 13,240 (82%) | 6,198 / 7,520 (82%) | 1,486 / 1,811 (82%) | 3,175 / 3,909 (81%) | - |
| Dedicated Clinics, n / N (%) | 10,873 / 13,240 (82%) | 6,082 / 7,520 (81%) | 1,546 / 1,811 (85%) | 3,245 / 3,909 (83%) | - |
| Local Early Arthritis Pathway, n / N (%) | 11,457 / 13,240 (87%) | 6,506 / 7,520 (87%) | 1,597 / 1,811 (88%) | 3,354 / 3,909 (86%) | - |
| Musculoskeletal Ultrasound, n / N (%) | 12,753 / 13,240 (96%) | 7,239 / 7,520 (96%) | 1,793 / 1,811 (99%) | 3,721 / 3,909 (95%) | - |
| Musculoskeletal Ultrasound at Same Day, n / N (%) | 5,274 / 13,206 (40%) | 3,192 / 7,496 (43%) | 713 / 1,810 (39%) | 1,369 / 3,900 (35%) | - |
| Shared Care, n / N (%) | 12,878 / 13,240 (97%) | 7,304 / 7,520 (97%) | 1,751 / 1,811 (97%) | 3,823 / 3,909 (98%) | - |
| Phone Adviceline, n / N (%) | 13,078 / 13,145 (99%) | 7,447 / 7,487 (99%) | 1,767 / 1,775 (100%) | 3,864 / 3,883 (100%) | - |
| Emergency Access, n / N (%) | 6,675 / 13,145 (51%) | 3,942 / 7,487 (53%) | 786 / 1,775 (44%) | 1,947 / 3,883 (50%) | - |

Table S2b Hospital characteristic table

| Year/ Hospital Characteristic | 2018  N=164 | 2019  N=164 | 2020  N=164 | 2021  N=164 | 2022  N=164 | 2023  N=164 |
| --- | --- | --- | --- | --- | --- | --- |
| Number of WTE Consultant, Mean (SD) | 3.7 (2.1) | 3.7 (2.1) | 3.8 (2.2) | 3.9 (2.3) | 4 (2.3) | 4 (2.3) |
| Number of WTE SAS, Mean (SD) | 0.3 (0.5) | 0.3 (0.6) | 0.3 (0.5) | 0.3 (0.6) | 0.3 (0.6) | 0.3 (0.6) |
| Number of WTE Training Grade, Mean (SD) | 1.2 (1.3) | 1.2 (1.3) | 1.2 (1.3) | 1.2 (1.3) | 1.2 (1.3) | 1.2 (1.3) |
| Number of Rheumatology Nurses, Mean (SD) | 3.1 (1.8) | 3.2 (1.9) | 3.3 (1.9) | 3.4 (2.1) | 3.7 (2.1) | 3.7 (2.1) |
| Access Musculoskeletal Physio, n / N (%) | 137 / 164 (91.3%) | 136 / 164 (90.7%) | 140 / 164 (93.3%) | 137 / 164 (91.3%) | 138 / 164 (92%) | 137 / 164 (91.3%) |
| Access Podiatry, n / N (%) | 108 / 164 (72%) | 104 / 164 (69.3%) | 112 / 164 (74.7%) | 104 / 164 (69.3%) | 116 / 164 (77.3%) | 117 / 164 (78%) |
| Access Occupational Therapy, n / N (%) | 139 / 164 (92.7%) | 138 / 164 (92%) | 137 / 164 (91.3%) | 139 / 164 (92.7%) | 138 / 164 (92%) | 137 / 164 (91.3%) |
| Access Psychology, n / N (%) | 55 / 164 (36.7%) | 53 / 164 (35.3%) | 56 / 164 (37.3%) | 41 / 164 (27.3%) | 46 / 164 (30.7%) | 44 / 164 (29.3%) |
| Early Arthritis Pathway, n / N (%) | 107 / 164 (71.3%) | 108 / 164 (72%) | 112 / 164 (74.7%) | 120 / 164 (80%) | 127 / 164 (84.7%) | 126 / 164 (84%) |
| Dedicated Clinics, n / N (%) | 102 / 164 (68%) | 106 / 164 (70.7%) | 107 / 164 (71.3%) | 112 / 164 (74.7%) | 109 / 164 (72.7%) | 109 / 164 (72.7%) |
| Local Early Arthritis Pathway, n / N (%) | 113 / 164 (75.3%) | 115 / 164 (76.7%) | 122 / 164 (81.3%) | 117 / 164 (78%) | 129 / 164 (86%) | 129 / 164 (86%) |
| Musculoskeletal Ultrasound, n / N (%) | 143 / 164 (95.3%) | 143 / 164 (95.3%) | 140 / 164 (93.3%) | 145 / 164 (96.7%) | 144 / 164 (96%) | 144 / 164 (96%) |
| Musculoskeletal Ultrasound at Same Day, n / N (%) | 51 / 164 (34.5%) | 50 / 164 (33.8%) | 54 / 164 (36.5%) | 53 / 164 (35.8%) | 49 / 164 (33.1%) | 50 / 164 (33.8%) |
| Shared Care, n / N (%) | 142 / 164 (94.7%) | 146 / 164 (97.3%) | 149 / 164 (99.3%) | 147 / 164 (98%) | 148 / 164 (98.7%) | 147 / 164 (98%) |
| Phone Adviceline, n / N (%) | 144 / 164 (97.3%) | 143 / 164 (96.6%) | 145 / 164 (98%) | 145 / 164 (98%) | 145 / 164 (98%) | 145 / 164 (98%) |
| Emergency Access, n / N (%) | 68 / 164 (45.9%) | 68 / 164 (45.9%) | 73 / 164 (49.3%) | 71 / 164 (48%) | 71 / 164 (48%) | 71 / 164 (48%) |

Figure S1 Annual patterns of csDMARD strategy stratified by age and gender

Figure S2 Specific csDMARD and GC strategy stratified by age and gender in RA patients with csDMARDs

Figure S3 Regional variation of first-line csDMARD choice


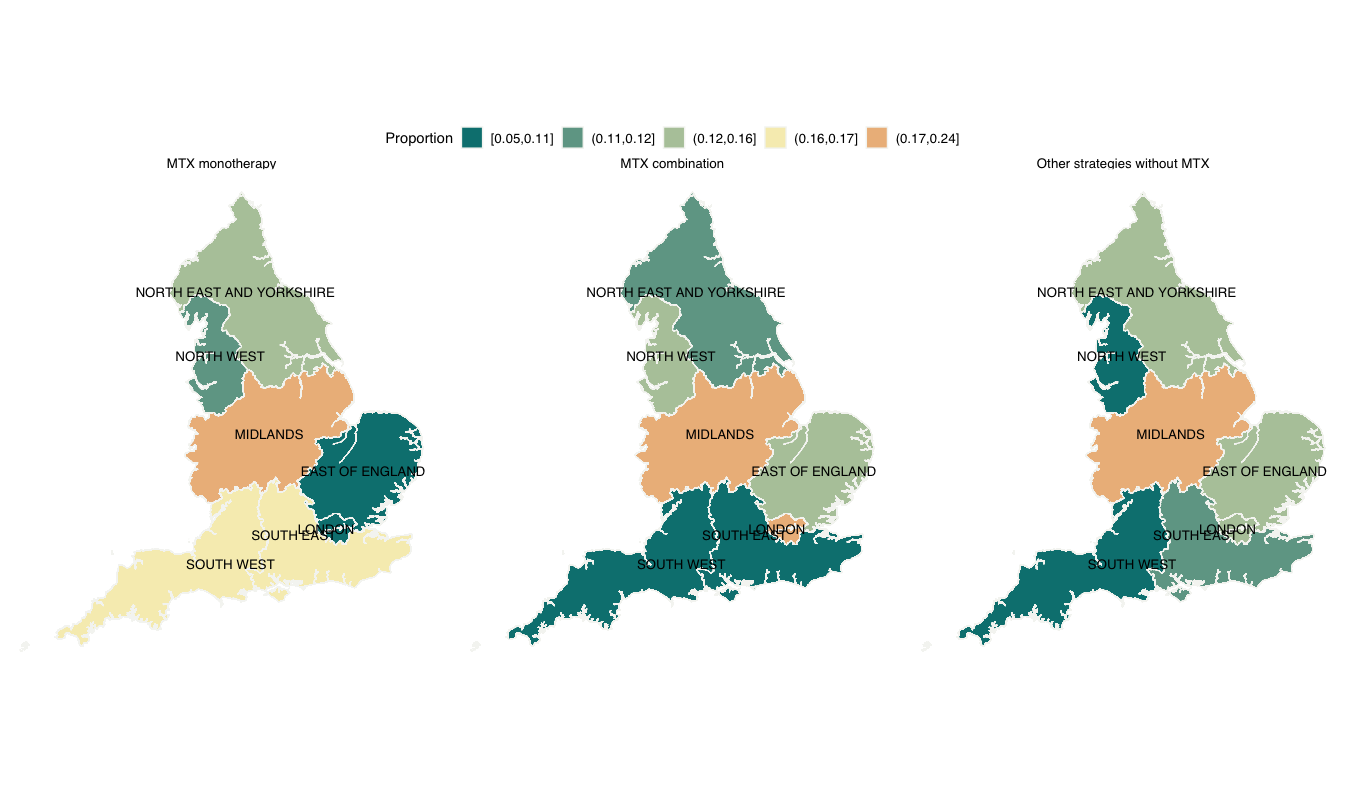


Figure S4 Regional variation of MTX and GC exposure in RA patients with csDMARDs


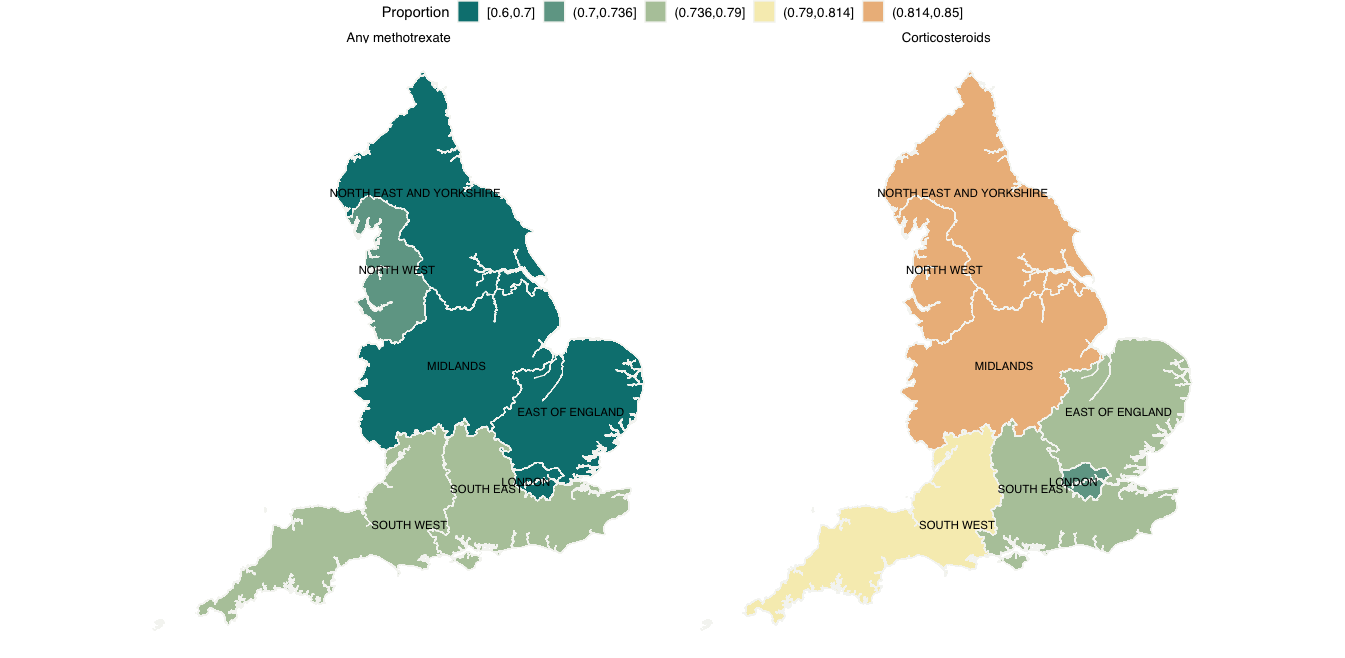


Figure S5 csDAMRD therapy exposure stratified by disease activity in RA patients with csDMARDs

Figure S6 Hospital variation among regions of MTX exposure in RA patients with csDMARDs


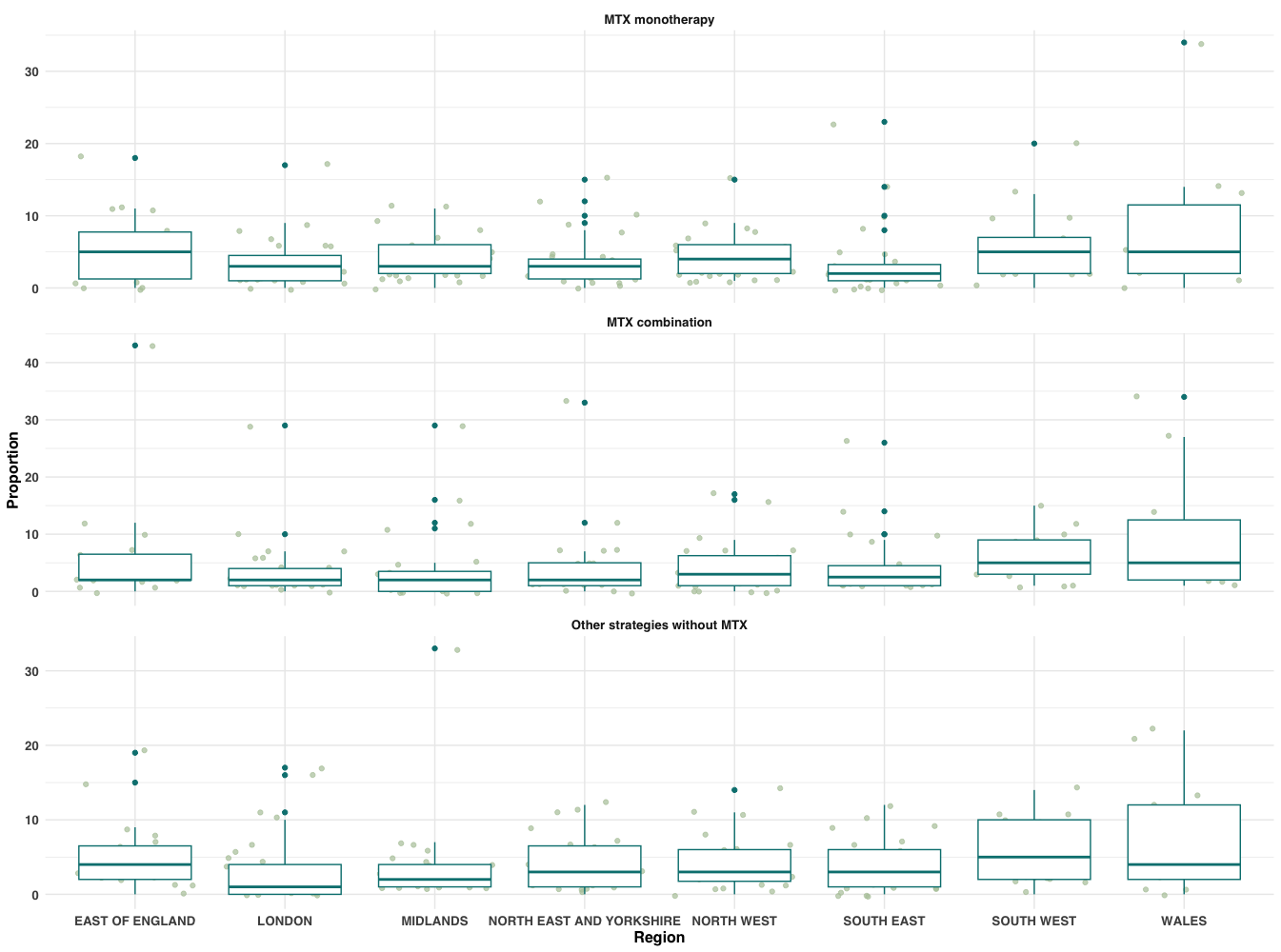


Table S3 Crude and adjusted odds ratios of patient-level factors with MTX compared with non-MTX treatment

| Characteristic | OR (MTX monotherapy) | 95%CI | aOR | 95%CI | OR (MTX combination) | 95%CI | aOR | 95%CI | OR (Any MTX) | 95%CI | aOR | 95%CI |
| --- | --- | --- | --- | --- | --- | --- | --- | --- | --- | --- | --- | --- |
| Age | 1 | 1 - 1.01 |  |  | 0.99 | 0.99 - 1 |  |  |  |  |  |  |
| <40 |  |  |  |  |  |  |  |  |  |  |  |  |
| 40-50 | 1.64 | 1.41 - 1.91 |  |  | 1.34 | 1.08 - 1.67 |  |  | 1.62 | 1.39 - 1.89 |  |  |
| 50-60 | 2.08 | 1.81 - 2.39 |  |  | 1.73 | 1.43 - 2.1 |  |  | 2.05 | 1.78 - 2.36 |  |  |
| 60-70 | 1.64 | 1.44 - 1.88 |  |  | 1.21 | 1 - 1.47 |  |  | 1.53 | 1.33 - 1.75 |  |  |
| 70-80 | 1.6 | 1.39 - 1.84 |  |  | 0.95 | 0.77 - 1.16 |  |  | 1.41 | 1.23 - 1.62 |  |  |
| >80 | 1.14 | 0.95 - 1.37 |  |  | 0.49 | 0.36 - 0.66 |  |  | 0.94 | 0.78 - 1.13 |  |  |
| Gender-Female | 0.83 | 0.76 - 0.9 |  |  | 0.93 | 0.83 - 1.04 |  |  | 0.84 | 0.77 - 0.92 |  |  |
| Ethnicity |  |  |  |  |  |  |  |  |  |  |  |  |
| White (ref) |  |  |  |  |  |  |  |  |  |  |  |  |
| Asian | 0.84 | 0.72 - 0.98 | 0.85 | 0.72 - 1 | 0.95 | 0.77 - 1.19 | 0.87 | 0.69 - 1.1 | 0.84 | 0.72 - 0.98 | 0.84 | 0.72 - 0.99 |
| Black | 0.68 | 0.52 - 0.88 | 0.74 | 0.56 - 0.98 | 0.97 | 0.7 - 1.33 | 0.94 | 0.67 - 1.33 | 0.78 | 0.6 - 1 | 0.78 | 0.61 - 1.01 |
| Mixed | 0.51 | 0.3 - 0.85 | 0.65 | 0.38 - 1.12 | 0.36 | 0.15 - 0.9 | 0.33 | 0.12 - 0.9 | 0.56 | 0.33 - 0.94 | 0.57 | 0.34 - 0.95 |
| Other | 0.95 | 0.76 - 1.2 | 1.01 | 0.79 - 1.28 | 0.91 | 0.65 - 1.29 | 0.81 | 0.56 - 1.17 | 0.96 | 0.76 - 1.21 | 0.96 | 0.76 - 1.22 |
| Smoking status |  |  |  |  |  |  |  |  |  |  |  |  |
| Never smoked (ref) |  |  |  |  |  |  |  |  |  |  |  |  |
| Current smoker | 1.02 | 0.92 - 1.14 | 1.01 | 0.9 - 1.13 | 1.12 | 0.96 - 1.3 | 1.08 | 0.92 - 1.27 | 1.06 | 0.95 - 1.18 | 1.02 | 0.92 - 1.14 |
| Ex-smoker | 0.94 | 0.85 - 1.03 | 0.92 | 0.83 - 1.02 | 0.87 | 0.76 - 0.99 | 0.91 | 0.78 - 1.05 | 0.95 | 0.86 - 1.04 | 0.92 | 0.83 - 1.01 |
| In paid work | 1.16 | 1.07 - 1.25 | 1.26 | 1.15 - 1.39 | 1.41 | 1.26 - 1.58 | 1.32 | 1.15 - 1.51 | 1.22 | 1.12 - 1.32 | 1.28 | 1.16 - 1.4 |
| Baseline DAS28 | 1.2 | 1.16 - 1.23 | 1.2 | 1.17 - 1.24 | 1.4 | 1.35 - 1.46 | 1.45 | 1.38 - 1.52 | 1.25 | 1.21 - 1.28 | 1.25 | 1.22 - 1.29 |
| Duration of symptoms |  |  |  |  |  |  |  |  |  |  |  |  |
| <1 month (ref) |  |  |  |  |  |  |  |  |  |  |  |  |
| 1-3 months | 1 | 0.86 - 1.17 | 1.03 | 0.87 - 1.21 | 1.09 | 0.87 - 1.36 | 1.14 | 0.9 - 1.45 | 1.06 | 0.9 - 1.23 | 1.05 | 0.9 - 1.23 |
| 3-6 months | 1.01 | 0.86 - 1.19 | 1.05 | 0.88 - 1.24 | 1.23 | 0.98 - 1.55 | 1.33 | 1.04 - 1.7 | 1.11 | 0.94 - 1.3 | 1.11 | 0.94 - 1.31 |
| 6-12 months | 1 | 0.84 - 1.18 | 1.06 | 0.89 - 1.27 | 1.05 | 0.82 - 1.34 | 1.13 | 0.87 - 1.46 | 1.07 | 0.91 - 1.27 | 1.08 | 0.91 - 1.28 |
| 1-5 years | 0.82 | 0.69 - 0.98 | 0.86 | 0.71 - 1.03 | 1.02 | 0.79 - 1.31 | 1.08 | 0.83 - 1.42 | 0.91 | 0.76 - 1.08 | 0.91 | 0.76 - 1.08 |
| >5 years | 0.89 | 0.67 - 1.19 | 0.9 | 0.66 - 1.23 | 0.85 | 0.55 - 1.32 | 0.97 | 0.61 - 1.54 | 0.92 | 0.69 - 1.24 | 0.92 | 0.69 - 1.24 |
| Comorbidity |  |  |  |  |  |  |  |  |  |  |  |  |
| Lung disease | 0.43 | 0.38 - 0.48 | 0.41 | 0.36 - 0.47 | 0.43 | 0.36 - 0.52 | 0.47 | 0.38 - 0.56 | 0.43 | 0.39 - 0.49 | 0.42 | 0.37 - 0.47 |
| Heart attack | 0.89 | 0.75 - 1.05 | 0.8 | 0.67 - 0.96 | 0.68 | 0.53 - 0.88 | 0.68 | 0.52 - 0.9 | 0.82 | 0.69 - 0.97 | 0.77 | 0.65 - 0.92 |
| Hypertension | 1.05 | 0.95 - 1.15 | 1.01 | 0.9 - 1.12 | 0.79 | 0.69 - 0.91 | 0.85 | 0.72 - 0.99 | 0.98 | 0.89 - 1.08 | 0.96 | 0.87 - 1.07 |
| Fracture | 0.77 | 0.6 - 1 | 0.81 | 0.62 - 1.07 | 0.53 | 0.36 - 0.79 | 0.61 | 0.4 - 0.92 | 0.74 | 0.58 - 0.96 | 0.75 | 0.58 - 0.97 |
| Diabetes | 1.1 | 0.96 - 1.26 | 1.1 | 0.95 - 1.27 | 0.88 | 0.73 - 1.07 | 0.89 | 0.72 - 1.09 | 1.06 | 0.93 - 1.22 | 1.05 | 0.91 - 1.2 |
| Cancer | 0.87 | 0.72 - 1.06 | 0.84 | 0.68 - 1.03 | 0.69 | 0.51 - 0.92 | 0.77 | 0.56 - 1.05 | 0.83 | 0.68 - 1.01 | 0.82 | 0.67 - 1 |
| Stomach ulcer | 0.84 | 0.68 - 1.03 | 0.84 | 0.67 - 1.05 | 0.89 | 0.66 - 1.2 | 0.94 | 0.69 - 1.29 | 0.86 | 0.7 - 1.06 | 0.86 | 0.7 - 1.06 |
| Depression | 0.95 | 0.82 - 1.1 | 0.98 | 0.84 - 1.15 | 0.9 | 0.73 - 1.11 | 0.89 | 0.71 - 1.12 | 0.94 | 0.81 - 1.09 | 0.96 | 0.82 - 1.11 |
| Seropositive | 1.01 | 0.92 - 1.1 | 1.01 | 0.92 - 1.12 | 1.47 | 1.28 - 1.68 | 1.41 | 1.22 - 1.63 | 1.08 | 0.99 - 1.18 | 1.09 | 0.99 - 1.2 |
| Corticosteroids | 2.01 | 1.83 - 2.22 | 2 | 1.8 - 2.22 | 2.45 | 2.1 - 2.86 | 2.65 | 2.24 - 3.13 | 2.13 | 1.93 - 2.36 | 2.13 | 1.93 - 2.36 |
| IMD |  |  |  |  |  |  |  |  |  |  |  |  |
| 1 (Least deprived,ref) |  |  |  |  |  |  |  |  |  |  |  |  |
| 2 | 1.03 | 0.83 - 1.28 | 1.12 | 0.88 - 1.42 | 1.04 | 0.76 - 1.43 | 1 | 0.71 - 1.41 | 1.1 | 0.87 - 1.38 | 1.1 | 0.88 - 1.38 |
| 3 | 0.93 | 0.75 - 1.16 | 0.98 | 0.77 - 1.25 | 0.9 | 0.66 - 1.23 | 0.89 | 0.63 - 1.24 | 0.95 | 0.76 - 1.2 | 0.95 | 0.76 - 1.2 |
| 4 | 1.02 | 0.8 - 1.3 | 1.06 | 0.82 - 1.37 | 1.06 | 0.75 - 1.49 | 0.96 | 0.66 - 1.39 | 1.03 | 0.8 - 1.32 | 1.04 | 0.81 - 1.33 |
| 5 | 1.01 | 0.81 - 1.28 | 1.04 | 0.81 - 1.33 | 0.96 | 0.7 - 1.32 | 0.91 | 0.65 - 1.28 | 1 | 0.79 - 1.27 | 1 | 0.79 - 1.27 |
| 6 | 0.97 | 0.76 - 1.23 | 1.04 | 0.8 - 1.36 | 0.94 | 0.66 - 1.33 | 0.87 | 0.6 - 1.26 | 0.98 | 0.76 - 1.25 | 0.98 | 0.76 - 1.26 |
| 7 | 1.09 | 0.85 - 1.39 | 1.17 | 0.9 - 1.52 | 1.08 | 0.76 - 1.53 | 1.09 | 0.75 - 1.57 | 1.13 | 0.88 - 1.45 | 1.14 | 0.88 - 1.46 |
| 8 | 0.94 | 0.74 - 1.2 | 1.06 | 0.81 - 1.39 | 1.05 | 0.73 - 1.51 | 1.04 | 0.7 - 1.56 | 1.05 | 0.81 - 1.36 | 1.05 | 0.81 - 1.36 |
| 9 | 0.96 | 0.76 - 1.22 | 1.03 | 0.8 - 1.33 | 0.98 | 0.68 - 1.39 | 0.92 | 0.63 - 1.34 | 0.99 | 0.78 - 1.27 | 1 | 0.78 - 1.28 |
| 10 (Most) | 1 | 0.76 - 1.3 | 1.04 | 0.78 - 1.38 | 0.9 | 0.61 - 1.33 | 0.82 | 0.54 - 1.24 | 0.97 | 0.74 - 1.27 | 0.97 | 0.74 - 1.27 |
| Region |  |  |  |  |  |  |  |  |  |  |  |  |
| EAST OF ENGLAND |  |  |  |  |  |  |  |  |  |  |  |  |
| LONDON | 1.19 | 0.62 - 2.26 | 1.12 | 0.58 - 2.16 | 2.02 | 1 - 4.05 | 1.94 | 0.95 - 3.94 | 1.26 | 0.7 - 2.25 | 1.26 | 0.71 - 2.26 |
| MIDLANDS | 2.1 | 1.09 - 4.02 | 2.09 | 1.08 - 4.06 | 1.49 | 0.73 - 3.03 | 1.52 | 0.74 - 3.12 | 2.01 | 1.12 - 3.6 | 2 | 1.12 - 3.59 |
| North East and Yorkshire | 2.14 | 1.12 - 4.1 | 2.17 | 1.11 - 4.21 | 1.2 | 0.58 - 2.46 | 1.08 | 0.52 - 2.26 | 1.93 | 1.07 - 3.49 | 1.93 | 1.07 - 3.48 |
| North West | 1.85 | 0.96 - 3.58 | 1.87 | 0.95 - 3.66 | 2.25 | 1.1 - 4.59 | 2.28 | 1.1 - 4.71 | 2.06 | 1.14 - 3.74 | 2.06 | 1.14 - 3.73 |
| South East | 2.27 | 1.19 - 4.33 | 2.18 | 1.13 - 4.21 | 1.07 | 0.52 - 2.21 | 1 | 0.48 - 2.1 | 1.87 | 1.05 - 3.36 | 1.88 | 1.05 - 3.37 |
| South West | 3.2 | 1.58 - 6.5 | 3.17 | 1.53 - 6.53 | 1.02 | 0.47 - 2.23 | 1 | 0.45 - 2.22 | 2.61 | 1.37 - 4.95 | 2.61 | 1.37 - 4.95 |
| Wales | 1.75 | 0.8 - 3.85 | 1.72 | 0.77 - 3.84 | 2.1 | 0.89 - 4.96 | 2.13 | 0.89 - 5.09 | 2.25 | 1.11 - 4.56 | 2.26 | 1.11 - 4.58 |
| Assessment Waiting time | 1 | - | 1 | - | 1 | - | 1 | - | 1 | - | 1 | - |
| Treatment waiting time | 1 | - | 1 | - | 1 | - | 1 | - | 1 | - | 1 | - |
| Referral via EIA pathway | 1.02 | 0.92 - 1.12 | 1 | 0.9 - 1.11 | 0.81 | 0.7 - 0.93 | 0.78 | 0.68 - 0.91 | 0.95 | 0.86 - 1.05 | 0.95 | 0.86 - 1.05 |
| Prescribe at same day as diagnosis | 0.49 | 0.44 - 0.54 | 0.48 | 0.43 - 0.53 | 0.67 | 0.58 - 0.77 | 0.67 | 0.58 - 0.78 | 0.51 | 0.46 - 0.56 | 0.51 | 0.46 - 0.56 |
| Year of prescription | 1.01 | 0.98 - 1.03 | 1.01 | 0.98 - 1.03 | 0.85 | 0.82 - 0.89 | 0.85 | 0.82 - 0.88 | 0.97 | 0.94 - 0.99 | 0.97 | 0.96 - 0.98 |

aOR: odds ratio adjusted by age and gender; 95%:CI: confidence intervals

Comparator: csDMARD treatment without MTX

Table S4 Crude and adjusted odds ratios of patient-level factors with MTX monotherapy compared with MTX combination therapy

| Characteristic | OR (MTX monotherapy VS MTX combination) | 95%CI | aOR | 95%CI |
| --- | --- | --- | --- | --- |
| Age | 1.01 | 1 - 1.01 |  |  |
| <40 | 1.51 | 1.3 - 1.76 |  |  |
| 40-50 | 1.78 | 1.56 - 2.04 |  |  |
| 50-60 | 1.57 | 1.37 - 1.79 |  |  |
| 60-70 | 1.61 | 1.4 - 1.84 |  |  |
| 70-80 | 1.35 | 1.12 - 1.62 |  |  |
| >80 | 0.85 | 0.78 - 0.92 |  |  |
| Gender-Female | 1.01 | 1 - 1.01 |  |  |
| Ethnicity |  |  |  |  |
| White (ref) |  |  |  |  |
| Asian | 0.83 | 0.72 - 0.97 | 0.88 | 0.76 - 1.03 |
| Black | 0.72 | 0.56 - 0.92 | 0.75 | 0.58 - 0.96 |
| Mixed | 0.78 | 0.46 - 1.31 | 0.83 | 0.49 - 1.4 |
| Other | 1.03 | 0.82 - 1.29 | 1.07 | 0.86 - 1.34 |
| Smoking status |  |  |  |  |
| Never smoked (ref) |  |  |  |  |
| Current smoker | 1 | 0.9 - 1.11 | 0.98 | 0.88 - 1.09 |
| Ex-smoker | 1 | 0.91 - 1.09 | 0.95 | 0.86 - 1.04 |
| In paid work | 1.04 | 0.96 - 1.13 | 1.17 | 1.07 - 1.27 |
| Baseline DAS28 | 1.09 | 1.06 - 1.12 | 1.08 | 1.05 - 1.11 |
| Duration of symptoms |  |  |  |  |
| <1 month (ref) |  |  |  |  |
| 1-3 months | 0.98 | 0.85 - 1.14 | 0.99 | 0.85 - 1.15 |
| 3-6 months | 0.95 | 0.81 - 1.11 | 0.97 | 0.83 - 1.13 |
| 6-12 months | 1 | 0.85 - 1.17 | 1.02 | 0.87 - 1.2 |
| 1-5 years | 0.81 | 0.68 - 0.97 | 0.83 | 0.7 - 0.99 |
| >5 years | 0.9 | 0.67 - 1.2 | 0.91 | 0.68 - 1.21 |
| Comorbidity |  |  |  |  |
| Lung disease | 0.53 | 0.47 - 0.6 | 0.5 | 0.44 - 0.56 |
| Heart attack | 0.98 | 0.83 - 1.16 | 0.88 | 0.74 - 1.05 |
| Hypertension | 1.14 | 1.03 - 1.25 | 1.06 | 0.96 - 1.17 |
| Fracture | 0.96 | 0.74 - 1.24 | 0.92 | 0.71 - 1.2 |
| Diabetes | 1.2 | 1.05 - 1.37 | 1.14 | 1 - 1.3 |
| Cancer | 0.95 | 0.79 - 1.16 | 0.9 | 0.74 - 1.09 |
| Stomach ulcer | 0.89 | 0.73 - 1.09 | 0.86 | 0.7 - 1.05 |
| Depression | 0.97 | 0.84 - 1.13 | 1.01 | 0.88 - 1.17 |
| Seropositive | 0.9 | 0.82 - 0.98 | 0.92 | 0.84 - 1.01 |
| Corticosteroids | 1.63 | 1.47 - 1.8 | 1.6 | 1.45 - 1.77 |
| IMD |  |  |  |  |
| 1 (Least deprived,ref) |  |  |  |  |
| 2 | 1.11 | 0.89 - 1.38 | 1.11 | 0.89 - 1.39 |
| 3 | 1 | 0.8 - 1.26 | 1.01 | 0.81 - 1.26 |
| 4 | 1.05 | 0.83 - 1.34 | 1.07 | 0.84 - 1.36 |
| 5 | 1.05 | 0.83 - 1.31 | 1.06 | 0.85 - 1.33 |
| 6 | 1.07 | 0.84 - 1.37 | 1.09 | 0.86 - 1.39 |
| 7 | 1.11 | 0.87 - 1.42 | 1.14 | 0.89 - 1.45 |
| 8 | 1 | 0.78 - 1.28 | 1.03 | 0.8 - 1.31 |
| 9 | 1.02 | 0.81 - 1.3 | 1.05 | 0.83 - 1.33 |
| 10 (Most) | 1.07 | 0.82 - 1.4 | 1.1 | 0.84 - 1.43 |
| Region |  |  |  |  |
| EAST OF ENGLAND |  |  |  |  |
| LONDON | 0.87 | 0.45 - 1.7 | 0.9 | 0.46 - 1.76 |
| MIDLANDS | 1.78 | 0.91 - 3.5 | 1.79 | 0.91 - 3.52 |
| North East and Yorkshire | 2.12 | 1.08 - 4.17 | 2.12 | 1.08 - 4.18 |
| North West | 1.44 | 0.73 - 2.84 | 1.45 | 0.73 - 2.87 |
| South East | 2.23 | 1.14 - 4.36 | 2.26 | 1.16 - 4.43 |
| South West | 3.21 | 1.54 - 6.68 | 3.17 | 1.52 - 6.63 |
| Wales | 1.35 | 0.6 - 3.04 | 1.36 | 0.6 - 3.07 |
| Assessment Waiting time | 1 | - | 1 | - |
| Treatment waiting time | 1 | - | 1 | - |
| Referral via EIA pathway | 1.08 | 0.98 - 1.19 | 1.08 | 0.98 - 1.19 |
| Prescribe at same day as diagnosis | 0.53 | 0.48 - 0.58 | 0.53 | 0.48 - 0.58 |
| Year of prescription | 1.06 | 1.03 - 1.08 | 1.06 | 1.05 - 1.07 |

aOR: odds ratio adjusted by age and gender; 95%:CI: confidence intervals

Table S5 Crude and adjusted odds ratios of hospital-level factors with MTX compared with non-MTX treatment

| Variable | OR  (MTX monotherapy) | 95%CI | aOR | 95%CI | OR  (MTX combination) | 95%CI |  | aOR | 95%CI | OR  (Any MTX) | 95%CI | aOR | 95%CI |  |
| --- | --- | --- | --- | --- | --- | --- | --- | --- | --- | --- | --- | --- | --- | --- |
| Number of WTE Consultant | 1.01 | 0.97 - 1.07 | 1.02 | 0.97 - 1.07 | 0.93 | 0.86 - 0.99 |  | 0.93 | 0.87 - 1 | 0.98 | 0.93 - 1.03 | 0.98 | 0.94 - 1.03 |  |
| Number of WTE Training Grade | 0.99 | 0.92 - 1.07 | 0.99 | 0.92 - 1.07 | 1.06 | 0.97 - 1.17 |  | 1.05 | 0.96 - 1.16 | 1.05 | 0.96 - 1.15 | 1.06 | 0.97 - 1.17 |  |
| Number of Rheumatology Nurses | 1.03 | 0.98 - 1.08 | 1.02 | 0.97 - 1.07 | 1.01 | 0.95 - 1.08 |  | 1.01 | 0.94 - 1.08 | 1.03 | 0.98 - 1.07 | 1.02 | 0.97 - 1.07 |  |
| Access Musculoskeletal Physio | 0.74 | 0.59 - 0.93 | 0.75 | 0.6 - 0.94 | 0.82 | 0.6 - 1.12 |  | 0.83 | 0.6 - 1.14 | 0.77 | 0.62 - 0.94 | 0.77 | 0.62 - 0.96 |  |
| Access Podiatry | 0.87 | 0.74 - 1.01 | 0.86 | 0.74 - 1.01 | 0.99 | 0.79 - 1.25 |  | 0.99 | 0.78 - 1.25 | 0.91 | 0.79 - 1.05 | 0.89 | 0.76 - 1.04 |  |
| Access Occupational Therapy | 0.77 | 0.56 - 1.05 | 0.75 | 0.55 - 1.02 | 1.31 | 0.85 - 2.02 |  | 1.26 | 0.81 - 1.96 | 0.89 | 0.66 - 1.18 | 0.88 | 0.65 - 1.18 |  |
| Access Psychology | 0.79 | 0.69 - 0.9 | 0.78 | 0.68 - 0.9 | 1.28 | 1.06 - 1.56 |  | 1.26 | 1.03 - 1.53 | 0.91 | 0.8 - 1.03 | 0.88 | 0.77 - 1.01 |  |
| Early Arthritis Pathway | 1.17 | 0.98 - 1.4 | 1.15 | 0.96 - 1.37 | 0.88 | 0.69 - 1.12 |  | 0.85 | 0.66 - 1.08 | 1.1 | 0.93 - 1.3 | 1.07 | 0.91 - 1.27 |  |
| Dedicated Clinics | 0.9 | 0.73 - 1.1 | 0.88 | 0.72 - 1.07 | 0.91 | 0.68 - 1.21 |  | 0.87 | 0.65 - 1.17 | 0.87 | 0.72 - 1.05 | 0.86 | 0.7 - 1.04 |  |
| Local Early Arthritis Pathway | 0.96 | 0.8 - 1.15 | 0.94 | 0.78 - 1.12 | 0.89 | 0.68 - 1.16 |  | 0.85 | 0.65 - 1.12 | 0.91 | 0.76 - 1.08 | 0.91 | 0.77 - 1.09 |  |
| Access to Musculoskeletal Ultrasound | 1.51 | 1.15 - 2 | 1.47 | 1.11 - 1.95 | 2.63 | 1.46 - 4.75 |  | 2.54 | 1.4 - 4.61 | 1.56 | 1.19 - 2.03 | 1.55 | 1.18 - 2.05 |  |
| Access to Musculoskeletal Ultrasound at Same Day | 1.02 | 0.86 - 1.21 | 1.03 | 0.87 - 1.22 | 1.1 | 0.87 - 1.39 |  | 1.12 | 0.89 - 1.42 | 1.03 | 0.88 - 1.21 | 1.05 | 0.89 - 1.24 |  |
| Shared Care | 0.79 | 0.53 - 1.18 | 0.79 | 0.53 - 1.19 | 0.67 | 0.4 - 1.14 |  | 0.65 | 0.38 - 1.1 | 0.77 | 0.52 - 1.13 | 0.77 | 0.52 - 1.15 |  |
| Phone Adviceline | 0.67 | 0.34 - 1.34 | 0.67 | 0.34 - 1.34 | 0.88 | 0.31 - 2.49 |  | 0.86 | 0.3 - 2.44 | 0.67 | 0.34 - 1.29 | 0.69 | 0.35 - 1.34 |  |
| Emergency Access | 1.23 | 1.06 - 1.43 | 1.25 | 1.07 - 1.45 | 0.71 | 0.57 - 0.89 |  | 0.72 | 0.57 - 0.9 | 1.1 | 0.96 - 1.27 | 1.12 | 0.97 - 1.29 |  |

aOR: case-mixed adjusted by age, gender, ethnicity, and DAS28 baseline

Comparator: csDMARD treatment without MTX
